# Recovery of Fertility in Azoospermia Rats after Injection of Adipose-Tissue-Derived Mesenchymal Stem Cells: The Sperm Generation

**DOI:** 10.1155/2013/529589

**Published:** 2013-02-18

**Authors:** Cihangir Cakici, Bugra Buyrukcu, Gokhan Duruksu, Ahmet Hakan Haliloglu, Ayca Aksoy, Ayca Isık, Orhan Uludag, Huseyin Ustun, Cansu Subası, Erdal Karaoz

**Affiliations:** ^1^Ankalife IVF and Women Health Centre, Ankara, Turkey; ^2^Stem Cell Department, Institute of Health Sciences, Kocaeli University Center for Stem Cell and Gene Therapies Research and Practice, Izmit, 41380 Kocaeli, Turkey; ^3^Urology Department, Faculty of Medicine, Ufuk University, Ankara, Turkey; ^4^Department of Pharmacology, Faculty of Pharmacy, Gazi University, Ankara, Turkey; ^5^Pathology Department, Ankara Hospital, Ankara, Turkey

## Abstract

The recent reports on the treatment of azoospermia patients, in which spermatozoa could not be traced in their testes, are focused more on the potential use of adult stem cells, like mesenchymal stem cells (MSCs). The aim of this study was to demonstrate the potential use of MSCs derived from adipose tissue in the treatment of azoospermia using rat disease models. After busulfan application, the rats (*n* = 20) were injected with the GFP^+^ MSCs into left rete testes. After 12 weeks, the testes with cell injection (right testes) were compared to control (left testes) after dimensional and immunohistochemical analyses. Testes treated with MSCs appeared morphologically normal, but they were atrophic in rats without stem cell treatment, in which the seminiferous tubules were empty. Spermatogenesis was detected, not in every but in some tubules of cell-treated testes. GFP^+^/VASA^+^ and GFP^+^/SCP1^+^ cells in testes indicated the transdifferentiation of MSCs into spermatogenetic cells in the appropriate microenvironment. Rats with cell treatment were mated to show the full recovery of spermatogenesis, and continuous generations were obtained. The expression of GFP was detected in the mesenchymal stem cells derived from adipose tissue and bone marrow and also in the sperms of offspring. In conclusion, MSCs might be studied for the same purpose in humans in future.

## 1. Introduction

The self-renewal and the multilineage differentiation capacities of adult stem cells (ASCs) show great promises for regenerative medicine. Despite of the greater differentiation potential of embryonic stem cells (ESCs) compared to ASCs, ethical concerns and governmental restrictions are the main obstacles of the ESCs standing in the way of their clinical applications [1]. On the other hand, bone-marrow-derived MSCs (BM-MSCs) are among the mostly studied ASCs, and their potential to treat a wide variety of diseases, including erectile dysfunction and male infertility, was demonstrated. Alternatively, adipose-tissue-derived MSCs (AT-MSCs) could be used in future clinical applications instead of bone marrow stem cells due to their comparable differentiation and therapeutic potential, but AT-MSCs are easier and safer to obtain [1–18].

The stem cells were relatively lately adapted in andrology researches on erectile dysfunction and infertility as potential therapeutic agents. The studies related in this area showed that ESC could participate in spermatogenesis by forming functional male germ cells *in vitro* or by supporting the maturation of primordial germ cells into haploid male gametes [19–21]. Nayernia et al. reported germ cell line formation from pluripotent teratocarcinoma cells in 2004, and after two years, the generation of offspring mice from ESC-derived germ cells was succeeded for the first time [22, 23]. The milestone in adult stem cell research to treat the infertility was the murine BM-MSC differentiation into male germ cells that was succeeded by the same group in 2006 [24]. The differentiation of BM-MSCs into germ cells, Sertoli cells, and Leydig cells was demonstrated in busulfan-treated infertile mice [25, 26]. MSCs derived from human fetal lung and umbilical cord were also shown to differentiate into sperm like cells [27, 28]. Due to their germ cell formation capacity *in vitro*, they suggested that those cells could be the base for a treatment of male infertility. These recent studies reveal that the treatment of diseases, like male infertility and testosterone deficiency, was possible by ASCs.

In this study, the mesenchymal-stem-cell-based therapy for azoospermia was aimed. For this purpose, allogenic AT-MSCs were injected into testicles of azoospermia rat models to recover the infertility. A strategy was herewith proposed for the treatment of azoospermia by intratesticular transfer of adult stem cells.

## 2. Materials and Methods

### 2.1. Animals

Animals were housed in the Laboratory Animal Care Center (Kobay A.S., Ankara, Turkey). All animal procedures were approved by the local ethical animal research committee. Male Wistar rats (*n* = 32) aged 8–12 weeks were housed in temperature-controlled rooms (20–22°C) under 12 h light/dark cycle. Later, female Wistar rats (*n* = 24) aged 8–16 weeks were housed for mating. The rats were fed with standard commercial chow diet *ad libitum*.

### 2.2. Experimental Design

MSCs were isolated from rat (*n* = 8) adipose tissue and labeled with GFP. The rest of male rats (*n* = 24) were sterilized with busulfan. After assessing the infertile status by analyzing the testes of rats (*n* = 4), the right testis of each rat (*n* = 20) was injected with MSCs. The other testis was left as control. After twelve weeks, testes of four animals were removed for dimension analysis. For immunohistochemical analyses, four additional rats were excised. The remaining male rats (*n* = 12) were mated with female rats (*n* = 24). Cells from offspring were analyzed for GFP expression.

### 2.3. Isolation and Culture of Rat Adipose-Tissue-Derived Mesenchymal Stem Cells (rAT-MSCs)

Rats (*n* = 8) were anesthetized by injection of 10 mg/kg Xylazine and 75 mg/kg Ketamine. 1-2 cm³ of preperitoneal adipose tissue was removed. Tissue samples were washed several times with Hanks' balanced salt solution supplemented with 5% antibiotic-antimycotic solution (Gibco Life Technologies, Paisley, UK), and vascular structures were removed. The yellowish white tissue was minced and enzymatically digested in MEM medium (Gibco Life Technologies) containing 0.075% collagenase 2 (Sigma, St. Louis, MO) at 37°C for 60 min. The cell suspension was filtered with 70 *μ*m sieve (Becton Dickinson Labware, Franklin Lakes, NJ). The cells were resuspended in MEM medium supplemented with 1% penicillin/streptomycin and 15% FBS (standard culture medium). After the centrifugation at 1200 rpm for 10 min, the cells were cultured in standard culture medium in 25 cm^2^ culture flasks. After 7 days, the medium was replaced with fresh medium and subsequently replaced twice a week. Erythrocytes and other non adhesive cells were removed from the culture. After reaching 70–80% confluence, the cells were harvested with 0.025% trypsin-EDTA for 3 min, collected by centrifugation, and subcultured at 1 : 3-1 : 4 ratio. The cells were counted by Trypan blue (Biological Industries, Kibbutz Beit Haemek, Israel). The blue staining of cells after mixing (1 : 1) was used as indicator of cell death.

### 2.4. Flow Cytometry

Undifferentiated rAT-MSCs were subjected to flow cytometry analysis. After passage 3 (P3), stem cells were harvested. Flow cytometry was performed using a FACS Calibur (BD Biosciences, San Jose, CA). Immunophenotyping analysis was performed against the following antigens: CD29, CD45, CD54, CD90, and CD106 (BD Biosciences). 

### 2.5. Immunostaining of rAT-MSCs

For immunofluorescence staining, samples were rinsed briefly in PBS and fixed in ice-cold methanol for 10 min. After permeabilization with 0.025% Triton X-100 (Merck, Darmstadt, Germany), the cells were incubated with 1.5% blocking serum (Santa Cruz Biotechnology, Heidelberg, Germany) in PBS for 30 min at 37°C followed by incubation overnight at 4°C with the primary antibody. After three PBS washes, cells were incubated with secondary antibodies for 25 min. The samples were mounted with mounting medium containing DAPI (Santa Cruz Biotechnology).

Immunohistochemical (IHC) analyses were performed using the streptavidin-peroxidase method (UltraVision Plus Detection System, Thermo Scientific, Chesire UK). Cultured cells were fixed in ice-cold methanol with 0.3% hydrogen peroxide (Carlo Erba) for 15 min. Cells were incubated with Ultra V Block for 5 min and incubated overnight at 4°C with the primary antibodies and for 15 min with secondary antibody at room temperature. After treatment with streptavidin peroxidase for 15 min at room temperature, signals were detected by the AEC kit (Zymed Laboratories, Inc., San Francisco, CA). The cells were counter-stained with hematoxylin (Santa Cruz Biotechnology). The list of primary antibodies was given in Table 1.

### 2.6. *In Vitro* Differentiation

To induce adipogenic differentiation, cells were seeded onto 6-well plates (P3; 3000 cells/cm^2^) and cultured with Mesencult MSC Basal Medium supplemented with 10% adipogenic supplement (Stem Cell Technologies Inc., Vancouver, BC, Canada) and 1% penicillin/streptomycin for 3 weeks. The medium was refreshed every 2–4 days. Intracellular lipid droplets indicate adipogenic differentiation confirmed by Oil Red O staining (0.5% in methanol; Sigma-Aldrich).

For osteogenic differentiation, cells (P3; 3000 cells/cm^2^) were seeded onto collagen I precoated cover slips in 6-well plates. The differentiation medium (MEM supplemented with 0.1 *μ*M dexamethasone (Sigma-Aldrich), 0.05 *μ*M ascorbate-2-phosphate (Wako Chemicals, Richmond, VA, USA), 10 mM *β*-glycerophosphate (Sigma-Aldrich), 1% antibiotic/antimycotic, and 10% FBS) was replaced twice a week. After four weeks, osteogenic differentiation was assessed by Alizarin red staining. For Alizarin red staining, cells were fixed for 5 min in ice-cold 70% ethanol. The cells were stained with Alizarin red solution (2%, pH 4.2) for 30 s. Stained cells were dehydrated in pure acetone, fixed in acetone-xylene (1 : 1) solution, and cleared with xylene. 

To induce neurogenic differentiation, cells (P3) seeded on collagen-I-coated cover slips were cultivated until 70% confluency. Cells were cultured for 3–5 days in differentiation medium (MEM supplemented with 0.5 mM isobutylmethylxanthine (IBMX), 10 ng/mL brain-derived neurotrophic factor (BDNF), 10 ng/mL epidermal growth factor (EGF), 10 ng/mL basic fibroblast growth factor (bFGF), 20% neural stem cell proliferation supplements (Stem Cell Technologies Inc.) and 1% penicillin-streptomycin). Differentiation was assessed by immunofluorescence staining of GFAP (SC-71141), HNK (SC-49034), Neurofilament (SC-12980), beta-tubulin (SC-9935), beta3-tubulin (SC-69965), c-Fos (SC-52), Nestin (SC-23927), and Eno2 (SC-59538). All antibodies were obtained from Santa Cruz Biotechnology. 

### 2.7. *In Vitro* Tube Formation Assay

To show the angiogenic potential of rAT-MSCs, tube formation was induced by culturing on Matrigel (Basement Membrane Matrix, LDEV-Free, BD Biosciences, Cat. No. 354234). 100 *μ*L of Matrigel was spread on the prechilled surface of each 24 well of the plate and incubated in standard culture medium (1 : 1) at 37°C for at least 30 min for polymerization. Cells suspended in serum supplemented medium were seeded on Matrigel-coated wells to a final density of 2.5 × 10^4^ cells/cm^2^. The formation of tube-like structures was observed after 24 h.

### 2.8. GFP Labeling of rAT-MSCs

The plasmid was supplied from Clontech (Palo Alto, CA), amplified in *E. coli* strain XL-1, and purified by Endofree PlasmidMaxi kit (Qiagen, Hilden, Germany). GFP coding gene was located downstream of the CMV (murine leukemia virus) constitutive promoter on vector. The cells were transfected by Neon Transfection System (Invitrogen Life Technologies, Carlsbad, CA) with the following parameters: 990 V, 40 ms, and 2 pulses. 2 × 10^5^ cells were mixed with 1 *μ*g plasmid DNA in 10 *μ*L transfer buffer. The transformed cells were cultured in MEM-medium supplied with 15% FBS. After 48 h, the cells were selected for antibiotic resistance toward G418 (400 *μ*g/mL) for 6 weeks. Then, the GFP stability of cells was monitored by continuous culturing for 4 passages, and the number of GFP^+^ cells was counted in flow cytometer. The integration of GFP gene into genome was checked by Real-Time PCR, and the copy number of integration in chromosome was determined.

### 2.9. Busulfan Treatment of Rats and Cell Transplantation

For long-term infertility, rats (*n* = 24) were injected with alkylating agent, busulfan (15 mg/kg; Sigma-Aldrich), twice with 14 days of interval to disrupt spermatogenesis. Once every 4 weeks, gonadotropin-releasing hormone (GnRH) agonist, leuprolide acetate (Lucrin, Abbott AS, Istanbul, Turkey), was administered subcutaneously (1.5 mg/rat) for 12 weeks until the animal was analyzed, according to the previous reports [29]. The effectiveness of this process was determined first by measuring testis size and weight. To confirm the effect, testes of 4 rats were removed, fixed in Bouin's fluid (9% formaldehyde, 5% acetic acid, 0.9% picric acid in water) and embedded in paraffin. To evaluate the spermatogenetic activity in tubules by histological analysis, the sections were stained with hematoxylin.

 AT-MSCs' suspension was mixed with sterile toluidine blue (1 : 1, v/v). Rete testis was identified by using stereomicroscope (Olympus, KL1500LCD), and these cells were injected into the lumen of the seminiferous tubules of recipient rat testis (*n* = 20), as described before [30]. 100 *μ*L of AT-MSCs' mixture (10^6^ cells) was injected into the rete testis of the left testicle (Figure 4) under stereomicroscope (Olympus, SZX7) using FemotoJet semiautomatic microinjector (Brinkmann Instruments Inc., Westbury, NY). The toluidine blue served as a marker to monitor the success of the injection. The untreated right testicle was served as control.

### 2.10. Analysis of Recipient Rats

Twelve weeks after cell transplantation, the testes of rats (*n* = 4) were analyzed dimensionally. The volume of testis was estimated by Cavalieri's principle. To localize the GFP-tagged rAT-MSCs in testes, rats (*n* = 4) were sacrificed for immune staining. Testis tissue samples were removed, fixed in formalin (10%, pH 7.0–7.6) for 24 h, and embedded in paraffin. Transversal serial sections (4 *μ*m thick) were taken. GFP labeled rAT-MSCs, used for cell tracking after injection, were double-stained on sections for GFP, and antigen of interest. Slides were deparaffinized with two changes of xylene for 5 min each and rehydrated in a series of graded alcohol solutions. Sections were antigen retrieved using a steamer-citrate buffer antigen retrieval method. Endogenous peroxidase was inhibited by incubation with fresh 3% H_2_O_2_ in PBS buffer. Nonspecific staining was blocked with the mixture of two different serums (with respect to the type of the antibodies used for blotting) in 1.5% PBS for 30 min at room temperature. Afterwards, the sections were incubated in the mixture of two primary antibodies in a pairwise fashion against GFP (SC-9996 or SC-5385) and vimentin (SC-7557), VASA (SC-67185), or SCP1 (SC-20837) for 1 h at RT. The sections were incubated in a mixture of two appropriate fluorescent-conjugated secondary antibodies and were mounted with mounting medium containing DAPI (Santa Cruz).

### 2.11. Mating the Rats

Female rats (*n* = 24) were mated during the proestrus phase with male rats (*n* = 12) with rAT-MSCs' transplantation. Every male rat was cohabitated with two female rats in polycarbonate cages until evidence of mating, vaginal plug, was observed. 

### 2.12. Detection of GFP Gene in Rat Chromosomal DNA

To determine whether these injected cells contributed in spermatogenesis and support the formation of offspring, GFP gene was traced in the genome of rat offspring. For this purpose, chromosomal DNA was isolated by QIAamp DNA Blood Mini Kit (Qiagen) from the blood samples of rat offspring according to the kit procedures. The GFP gene was amplified first by conventional PCR using the following primer pairs: 5′-cttgttgaattagatggtgatg/5′-ctgttacaaactcaagaaggacc. Template DNA-free PCR reaction was used as negative control. The gene copy number in DNA samples was estimated by real-time PCR using the same primer pairs and Power SYBR Green Master Mix (Applied Biosystems Life Technologies) in amplification reaction. Sox2 gene was amplified as reference for each DNA sample with the following primer pairs: 5′-atgtacaacatgatggagacg/5′-tcacatgtgtgagaggggcagtg. The GFP gene in genome was further confirmed by Southern blot hybridization assay [31]. 5 *μ*g of rat chromosomal DNA was incubated with XbaI and XhoI restriction enzymes (10 U/each; Fermentas, Vilnius, Lithuania) for 16 h at 37°C, blotted on positively charged nylon membrane (Roche) by capillary action, and detected by DIG (digoxigenin) Nucleic Acid Detection Kit (Roche) according to the protocol by manufacturer. Oligonucleotide probe for detection was synthesized by random labeling of GFP gene with DIG-labeled nucleotides. The detection of chemiluminescence was performed by DNR Bio-Imagining Systems (MF-ChemiBIS 3.2, Jerusalem, Israel). 

### 2.13. Detection of GFP Expression in Offspring's rBM-MSCs and rAT-MSCs

To observe the GFP expression in MSCs of offspring, both adipose-tissue- and bone-marrow-derived MSCs were analyzed. rAT-MSCs were isolated and characterized by the methods described previously (Sections 2.3–2.6). rBM-MSCs were isolated with the following procedure. Under sterile conditions, both rat femur and tibiae were excised, and the bone marrow was extruded by flushing with standard culture medium using 21-gauge needle. Marrow plug suspension was dispersed by pipetting, successively filtered through 70 *μ*m mesh nylon filter (BD Biosciences, Bedford, MA), and centrifuged at 200 xg for 10 min. The bone marrow was diluted to 1 : 3 with PBS and layered over a Ficoll-histopaque gradient (1.077 g/mL, Sigma). The low-density mononuclear cells were collected, washed twice with PBS, counted, and plated in tissue culture flasks at a density of 1.4 × 10^5^ cells/cm^2^ in standard culture medium. The MSCs were isolated based on their ability to adhere on plastic. By replacing the culture medium with fresh medium, the unattached cells were removed from the culture, and the culture was allowed to grow further. Cells at 70–80% confluency were harvested with 0.25% trypsin-EDTA solution after washing with Ca^2+^-Mg^2+^-free PBS. Both rBM-MSCs and rAT-MSCs from offspring were cultured to passage 2 (P2), and immunofluorescence staining was performed as previously described in Section 2.5. To detect the GFP expression in stem cells, anti-GFP antibody was used.

Both rBM-MSCs and rAT-MSCs from offspring were cultured to passage 2 (P2), and immunofluorescence staining was performed as previously described. To detect the GFP expression in stem cells, anti-GFP antibody was used. 

To show the GFP^+^ sperms, the testes of offspring (1st generation) were removed and cut in small sizes in Ca^2+^-Mg^2+^-free Hank's Balanced Salt Solution (HBSS with, Gibco Life Technologies) with 0.35 g/L sodium bicarbonate and 1 g/L D-glucose. Large tissue particles were removed, and sperm cells were collected on glass slides in the cytocentrifuge (1500 rpm, 10 min). Slides were stained with antibody to GFP (Santa Cruz, sc-5385). The nuclei were labeled with DAPI.

### 2.14. Statistical Analysis

A computer program (SPSS 10.0) was used for statistical analysis. The results were expressed as means ± standard deviation (SD). Two-tailed paired *t*-test was used for the comparison of the groups. Differences between the groups were considered as statistically significant when *P* < 0.05 and highly significant when *P* < 0.01.

## 3. Results

### 3.1. Culture of rAT-MSC

**Figure 1:**
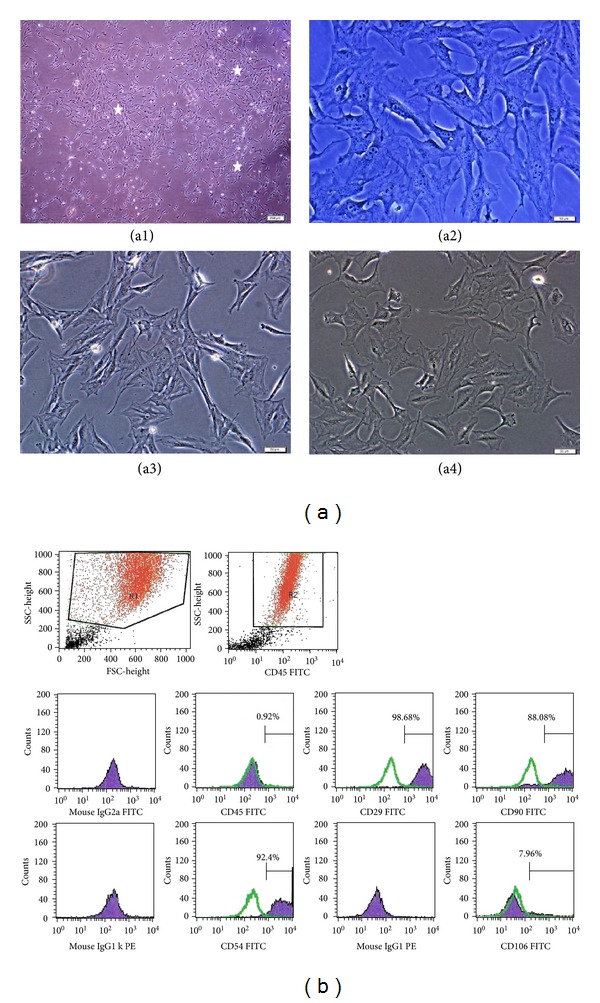
Morphological and phenotypic characteristics of rAT-MSCs. During the onset of culture (a1: P0-5th day), the isolated cells from rat adipose tissue formed single-cell-derived colonies (arrows). After the next days and passages, most of these SCs exhibited large, flattened or fibroblast-like morphology (a2: P0-7th day, a3: P1-2th day, and a4: P3-1th day). (b) A representative flow cytometry analysis of cell-surface markers of rAT-MSCs at P3; cells were labeled with antibodies against hematopoietic (CD45) and MSC markers (CD29, CD54 and CD90) and with vascular cell adhesion protein 1 (CD106). (green line: histogram of isotype control immunoglobulin).

MSCs attached to the culture flasks sparsely and displayed a fibroblast-like, spindle-shaped morphology during the initial days of incubation. Following 3-4 days of incubation, proliferation started and the cells gradually grew into small colonies. During culture, adjacent colonies interconnected with each other, and a monolayer confluence was obtained after 12–15 days of incubation. In later passages, MSCs exhibited large, flattened fibroblast-like morphology (Figures 1(a1)–1(a4)) and did not change throughout 25 passages. Tests for bacterial and mycoplasma contamination were negative. The viability of cells was higher than 95%, determined by Trypan blue staining of cells. rAT-MSCs expressed CD29, CD54, and CD90, but not CD45 and CD106 (Figure 1(b)) and maintained their phenotype in the following passages.

### 3.2. Immunostaining of rAT-MSCs

**Figure 2:**
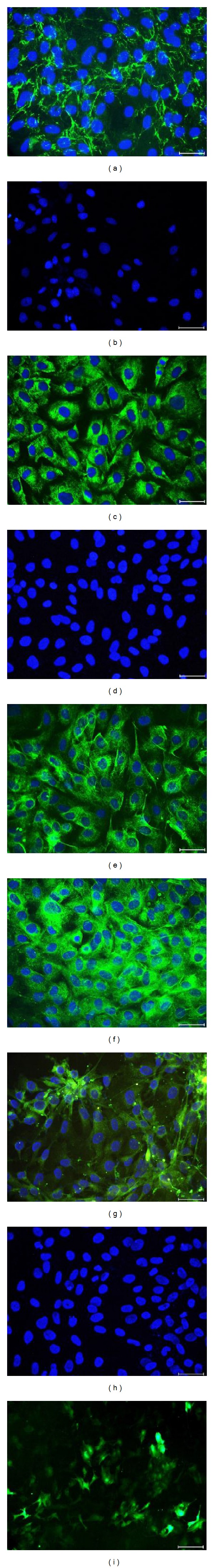
Immunofluorescence staining of rAT-MSCs for fibronectin (a), CD34 (b), GFAP (c), CD45 (d), Nestin (e), Vimentin (f), Map2a, b (g), and Cytokeratin 18 (h). Staining pattern was cytoplasmic for fibronectin, GFAP, nestin, and vimentin; and both membranous and cytoplasmic for Map2a, b. After transfection, rAT-MSCs showed GFP^+^ immunostaining (i). Nuclei were labeled with DAPI (blue). Scale bars: 50 *μ*m.

Immunohistochemical studies were performed to characterize the progeny of the rAT-MSCs by using antibodies specific to known antigens of MSCs. Immune reactivity profile for rAT-MSCs was shown in Table 1. Under standard culture conditions, fibronectin (Figure 2(a)), GFAP (Figure 2(c)), Nestin (Figure 2(e)), Vimentin (Figure 2(f)), Map2a, b (Figure 2(g)), CD105, Collagen type-I, Collagen type-II, beta-tubulin, ASMA, Myogenin, Osteopontin, Osteocalcin, Osteonectin, Ki67, PCNA, and Tenascin (data not shown) were expressed. Surface markers including CD34 (Figure 2(b)), CD45 (Figure 2(d)), Cytokeratin 18 (Figure 2(h)), and CD71 (Table 1) were not expressed by rAT-MSCs. 

### 3.3. Differentiation Potential of rAT-MSCs

**Figure 3:**
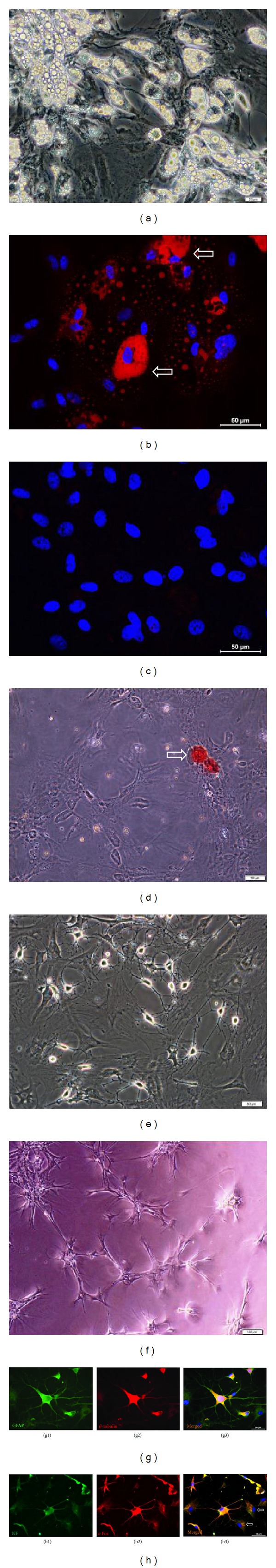
Microscopic images of rAT-MSCs differentiated into adipocytes (a, b), osteoblasts (d), neuron-glial like cells (e), and endothelial cells (f). The arrows indicate the neutral lipid vacuoles stained with Oil Red O (b). rAT-MSCs were undifferentiated in standard culture medium (c). Phase contrast microscope images of the rAT-MSCs differentiated into osteogenic lineage, where calcified nodules (arrow) were stained with Alizarin red S (d). Differentiation of rAT-MSCs to neuron-glial like cells after 3 days (e). Endothelial tube formation by rAT-MSCs on Matrigel (f). Immunostaining of cells for GFAP (green) and beta-tubulin (red) differentiated cells (g1–g3). Increased cytoplasmic and nuclear staining of differentiated cells for c-Fos (red) and NF (green; cytoplasmic and membranous) was observed (h1–h3).

rAT-MSCs (P3) were differentiated within 3 weeks in the adipogenic differentiation medium. In cells, lipid droplets enlarged and invaded the entire cytoplasm (Figures 3(a) and 3(b)). The lipid droplet formation was not observed in undifferentiated rAT-MSCs (Figure 3(c)).

In the osteogenic differentiation, cells proliferated and reached almost complete confluency after 8–10 days of incubation. Later, the cellular aggregates were observed in differentiated cultures and the gradually increased. The aggregates were characterized by the presence of amorphous material deposits. These nodular aggregates in osteogenic cultures were stained with Alizarin red S after 28 days, demonstrating that the amorphous deposits were actually calcium deposits (Figure 3(d)). 

rAT-MSC-derived neuron-like cells displayed distinct morphologies, ranging from extensively simple bipolar to large, branched multipolar cells (Figure 3(e)). Within 24 h, these stem cells formed tube-like structures after culturing on Matrigel (Figure 3(f)). For characterizing their neuronal character further, differentiated rAT-MSCs were stained for neuron and glial cell specific markers including GFAP (Figures 3(g1) and 3(g3)), beta-Tubulin (Figures 3(g2) and 3(g3)), Neurofilament (Figures 3(h1) and 3(h3)), c-Fos (Figures 3(h2) and 3(h3)), Map-2a,b, beta3-Tubulin, Eno2, and HNK (data not shown). 

### 3.4. Testicular Size and Spermatogenesis

**Figure 4:**
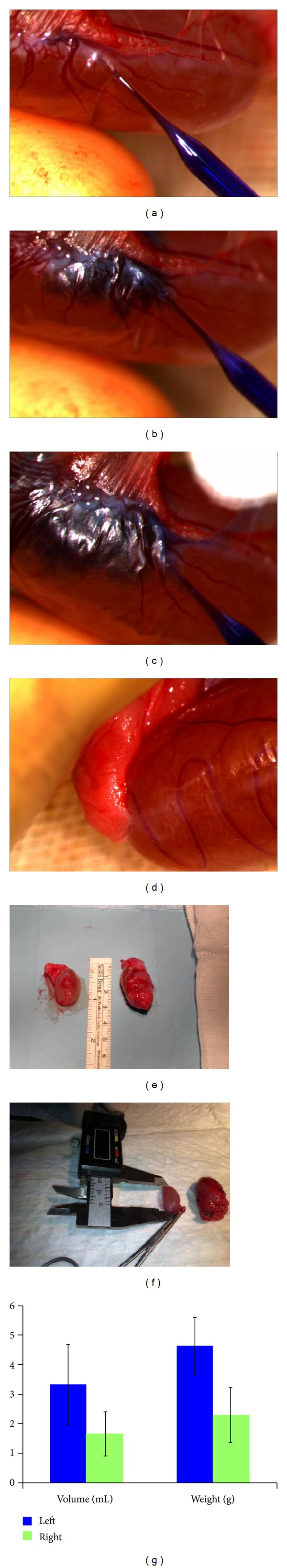
Cell transplantation into rete testis and morphological analyses of testes after 12 weeks. 1 × 10^6^ cells were mixed with toluidine blue (1 : 1, v/v), and the mixture was injected into the rete testis of the left testicle of rats (a–c). The right testicle of rats was left untreated after busulfan (d). After 12 weeks, the atrophy in right testicles (f) was significant, when compared with the left testicles with rAT-MSCs treatment (e). There were significant differences in both volume and weight of testicles: *P* = 0.0074 and *P* = 0.0015, respectively (g).

The difference in dimensions of the left (treated with rAT-MSCs) and the right testicles (without cell transplantation) were analyzed, and mass increase of almost 50% was measured in testes with rAT-MSCs (Figures 4(e), 4(f), and 4(g)). It was observed that the average volume of the testicles with rAT-MSCs was higher than the ones with no transplantation and atrophy was not seen in testicles with rAT-MSCs injection (Figures 4(e) and 4(f)). The volume increase of busulfan-treated testes was only observed significantly in the test subjects with MSCs transplantation (Figure 4(g)). A significant increase of size in other interstitial tissues was not noticed.

### 3.5. Histological Assessment of Spermatogenesis

**Figure 5:**
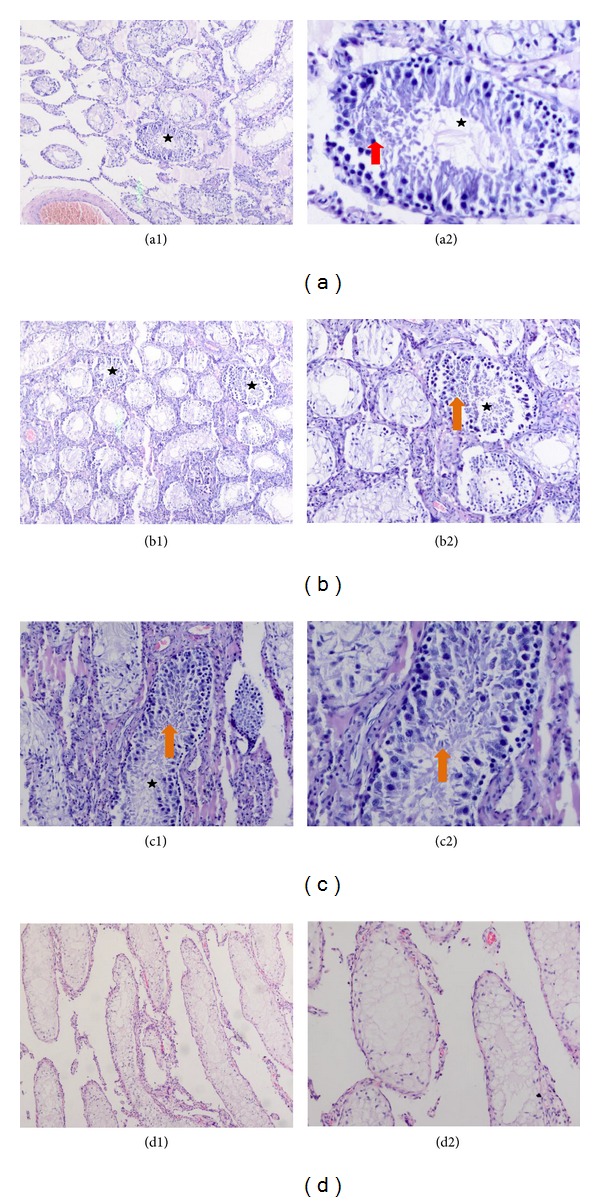
Sections of treated testes with rAT-MSCs. Most of the seminiferous tubules were empty indicating the absence of spermatogenesis. Only a few tubules appeared to be filled with spermatogenetic cells up (asterisks). Spermatogenetic cells were shown in these tubules (red arrow) (a2). Spermatozoa were also shown with arrows (orange arrows) (b1, b2, and c2). Sections of busulfan-treated testis (right), which are not transplanted with stem cells, demonstrate empty seminiferous tubules that indicate no spermatogenetic activity (d1, d2). The higher magnification of the slides (a1, b1, c1, and d1) was shown on the right of the same sections (a2, b2, c2, and d2). Original magnifications: a1, b1, d1-X48, c1, b2, d1-X100, a2, c2-X200.

Formalin-fixed and paraffin-embedded testis tissue sections were stained with hematoxylin-eosin. These samples from stem-cell-injected tissues were examined under light microscope for any spermatogenic activity. After generating the infertility in rat with double injection of 15 mg/kg of busulfan, the testes of the control animals were thoroughly analyzed for any spontaneous recovery of spermatogenesis, and no sign was observed for reinitiated spermatogenesis. The scanning of sections showed atrophy, complete, and incomplete spermatocytic arrest and Sertoli cell-only appearance for samples without rAT-MSCs. After the treatment with busulfan, spermatogenesis process was blocked (Figures 5(d1) and 5(d2)). However, the presence of spermatogonium in the tissues with stem cell transplantation was noted (Figures 5(a1)–5(c2)). The seminiferous tubules of the controls, which were not treated with MSCs, were empty and indicated the disruption of spermatogenesis (Figures 5(d1) and 5(d2)). On the other hand, the tubules appeared to be filled up with spermatogenetic cells in the sections of cell-treated tissues, but with low rate. Spermatozoa were observed (Figures 5(a1)–5(c2)).

### 3.6. Detection of Spermatogenic Cells and Markers for Meiosis

**Figure 6:**
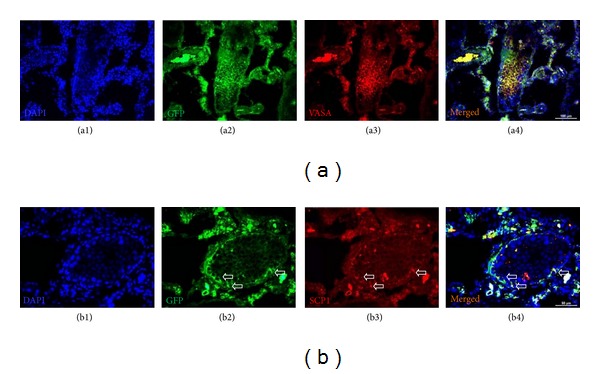
Immunostaining of GFP^+^/VASA^+^ and GFP^+^/SCP1^+^ cells in busulfan-treated testis with rAT-MSCs injection. GFP^+^/VASA^+^ cells were localized in the seminiferous tubules of testis (white star), but most of the tubules were empty (blue asterisk) (a1–a4). The expression of meiosis marker SCP1 in GFP^+^ cells might indicate the transdifferentiation of MSCs into spermatogenic cells (white arrows) (b1–b4).

The rAT-MSC-injected testis sections were positive for both GFP and VASA (Figures 6(a1)–6(a4) and 7). VASA-positive staining pointed to the presence of spermatogenic cells in tubules, and the GFP staining designated the origin of cells to be the rAT-MSCs used in transplantation. Interestingly, not all GFP^+^ cells were expressing VASA (germ cell marker). However, the GFP^+^ and VASA^+^ cells (yellow) might indicated that those cells possibly underwent in the sperm formation process in tubules. The expression of meiosis marker, SCP1, also supported the evidence of participation of rAT-MSC in spermatogenesis (Figures 6(b1)–6(b4)). Both markers were not expressed in the undifferentiated state of rAT-MSC in cell culture *in vitro* (see in Supplementary Material available on line at http://dx.doi.org/10.1155/2013/529589. Data 1). 

### 3.7. Restoration of Male Fertility and Birth of Offspring

**Figure 8:**
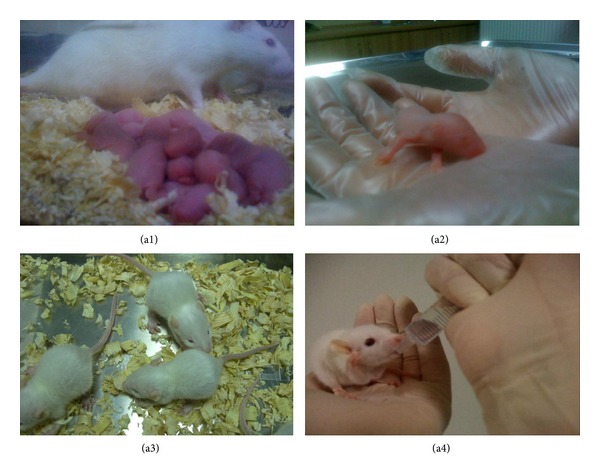
Newborns (offspring) after pairing healthy females with rAT-MSC-injected males with busulfan-treated testes. Offspring after birth (a1-a2) and after 10 days (a3-a4).

**Figure 9:**
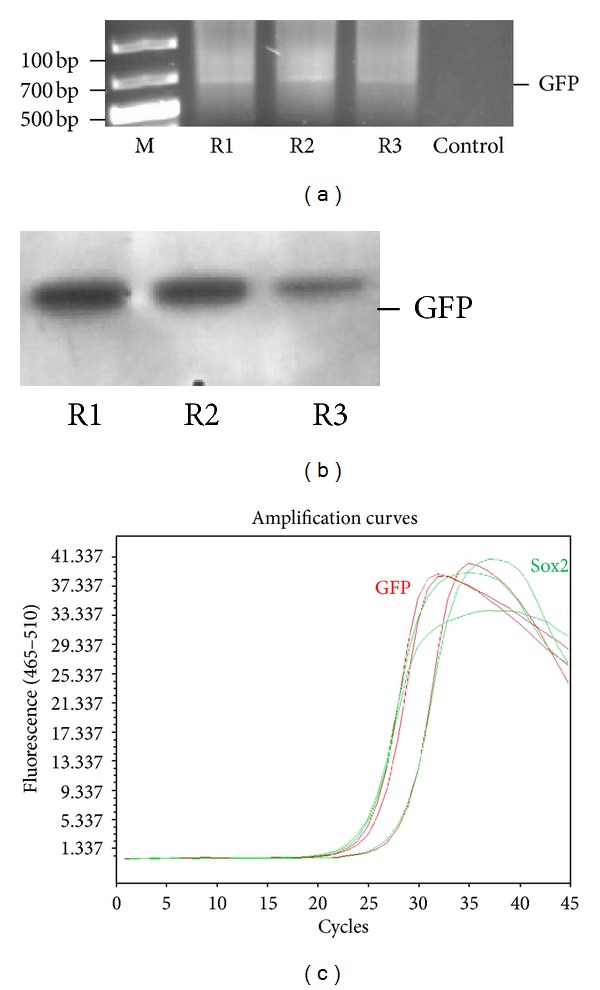
Representative analysis of chromosomal DNA for exogenous gene (GFP) insertion for three offspring (R1–R3). DNA isolated from blood samples were analyzed for GFP gene by PCR amplification (a) and southern blot hybridization (b). Gene copy number of GFP and Sox2 in genome was estimated to be same by Real-Time PCR (c). The GFP gene in chromosomal DNA of rat supported the evidence for transdifferentiation of MSCs into functional sperms.

**Figure 10:**
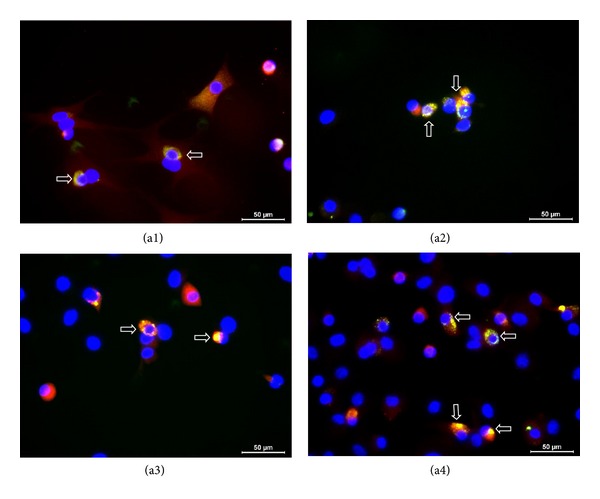
GFP^+^/Vimentin^+^ mesenchymal stem cells derived from bone marrow (P0) from offspring. rBM-MSCs were isolated from the offspring (a1–a4), and some vimentin- (red) positive cells showed the GFP (green) expression (arrows). The expression of nonmammalian gene GFP in rBM-MSCs indicates the transdifferentiation of GFP-labeled rAT-MSCs in testes.

**Figure 11:**
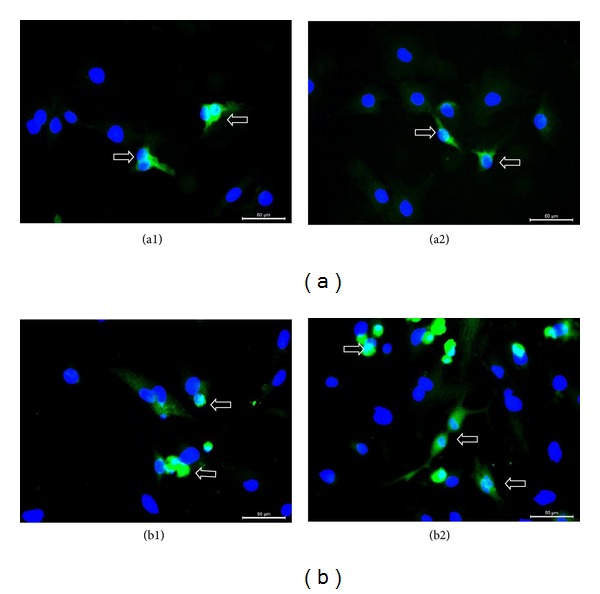
GFP^+^ rBM- and rAT-MSCs (P2) derived from offspring. MSCs were isolated and characterized from the bone marrow (a1-a2) and adipose tissue (b1-b2) of offspring. The GFP-gene could only be originated from the paternal rat injected with GFP labeled rAT-MSCs.

After mating and successful pregnancy periods following pairing of female rats with male rats with GFP-labeled rAT-MSCs, 9 viable offspring were obtained (Figures 8(a1)–8(a4)). These male rats were further mated with other females, and the preserved fertility was observed in stem-cell-transplanted animals by obtaining the next offspring. To determine whether the offspring were originated from stem-cell-treated males' spermatozoa, 0.4 mL of blood of each animal was collected for DNA isolation and detection of GFP gene in genome. GFP gene was detected by PCR and Southern blot hybridization (Figures 9(a) and 9(b)). The fragment of expected size (634 bp) was amplified in conventional PCR. Exogenous gene integration into genome of offspring was also shown by Southern blot hybridization for GFP (Figure 9(b)). Because the parental rats lack the GFP gene, the only possible origin of this foreign gene in offspring would be the GFP gene used to label rAT-MSCs prior to the injection in testes. The copy number of GFP gene was estimated to be equal to the reference gene, Sox2, by real-time PCR. The gene copy was also estimated in rAT-MSCs before transplantation to be the same as Sox2 gene (Figure 9(c)). The MSCs were isolated from the bone marrow and adipose tissue of the offspring (Figures 10 and 11). The immunostaining of those cells with GFP antibody gave positive result, meaning that both types of MSCs inherited their GFP gene from the paternal rats and were functionally expressed in their cytoplasm like the rAT-MSCs injected in testes. The location of the GFP expression was observed in cytoplasm, but very close to the nuclei. The same staining pattern was also observed in the cell culture of GFP^+^ rAT-MSCs (see Supplementary Data 2). The only possible source of this nonmammalian gene could be the GFP labeled rAT-MSCs, injected in infertile male rats, it could be considered as a significant evidence for the maturation of functional sperms from injected rAT-MSCs. In the analysis of GFP^+^ cells, MSCs were deliberately selected because of the complexity of mammalian gene expression regulation and being aware of GFP was previously expressed in MSCs. In addition, the presence of GFP was also detected in the sperms of offspring (see Supplementary Data 3). The sperm cells were stained with GFP antibody to increase the intensity of green light and their nuclei were shown by DAPI. The sperm cells were observed with GFP accumulation overlying the nuclei. This transgenic rat line has been propagated to the third generation by inbreeding without silencing of transgene expression.

## 4. Discussion

The development of stem-cell-based therapies currently represents one of the major challenges of medical research. Adipose-tissue-derived mesenchymal stromal cells could be obtained from the stromal vascular fraction [32] under *in vitro* culture conditions. These cells are characterized by fibroblast-like morphology, adherence to plastic surfaces, continuous cell proliferation over long culture periods and multilineage differentiation capacity. They could transdifferentiate into variety tissue cells including endothelial, epithelial, muscle, Schwann cells, hepatocytes and neurons *in vitro* [15, 32–42]. 

 In infertility and sterility, stem cell therapy promises to be a potential source of male and female germ cells. Not only ESCs but also fetal porcine skin stem cells, human fetal lung-MSCs, bone marrow, and umbilical cord MSCs were the candidate for the germ cell differentiation *in vitro *[20, 23, 25, 28, 43–46]. On the other hand, stem cells experimentally derived from bone marrow have been recently used in experimental busulfan-treated infertility rodent models. Nayernia et al. [23, 24] showed for the first time that murine BM-MSCs could differentiate into male germ cells. Yazawa et al. [47] proved that MSCs have the capacity to differentiate into steroidogenic cells, such as Leydig cells, both *in vivo* and *in vitro* [46]. More recently, Lue et al. [26] showed that BM-MSCs, transplanted into testis of a busulfan-treated infertility mouse model, appeared to differentiate into germ cells, Sertoli cells, and Leydig cells [26]. This finding raises the possibility of using MSCs to treat male infertility and testosterone deficiency. The general consensus on AT- and BM-MSC is that they are virtually identical in cell surface marker profile, gene expression profile, and differentiation potential [12]. This issue has been confirmed in a preclinical study in which both cells were found equally effective in treating a porcine model of cardiac infarction [17]. Whereas bone marrow could only be obtained in limited quantity, the adipose tissue is usually available in abundance. Therefore, the difference in the clinical application potential of AT-MSC and BM-MSC is quite obvious [1]. Numerous studies conducted to date have indicated that stem cells derived from adult human tissues could be reprogrammed to differentiate into different cell types. However, no progress or evidence has been reported so far in the isolation and characterization of AT-MSCs to differentiate into functional germ cells. This study showed the differentiation potential of clonally expanded MSCs into germ cells or sperm-like cells. The transplantation of AT-MSCs was successfully achieved into testis through rete testis of busulfan-treated infertility rat models. Later, there was observed the recovery of the fertile status of those males. The analyses also supported the evidences of the functional spermatogenesis progress in testes. 

The expression of GFP and the meiosis marker, SCP1, by these cells in tubules might indicate the involvement of rAT-MSC in spermatogenesis. The function of MSCs in spermatogenesis might be direct (transdifferentiation), or they interact with niche of testis tissue. Reprogramming might be achieved by changing the cellular microenvironment (niche), in which the cells grow, to provide signals that might activate appropriate metabolic pathways. Successful experiments were performed *in vivo* by using the microenvironment of the target cell type. When the conditions were provided similar to the *in vivo* microenvironment, differentiation of stem cells into the targeted cell types with full functionality could be obtained also *in vitro* [48–58]. In the study by Kim et al., it was indicated that the niche might include some factors that promote the genetic and epigenetic status of stem cell self-renewal *in vitro* [27]. Most importantly the niche was pointed out for serving as a cellular platform for the expansion of stem cells that could potentially be exploited for therapeutic tissue/organ neogenesis.

The entire process of spermatogenesis from spermatogonial stem cells (SSCs) to spermatozoa takes place in the seminiferous tubules. SSCs involve into regeneration and maintenance of spermatogenesis and are located in the basal compartment, also recognized as niche. This site was defined as specialized microenvironment necessary for maintenance of stem cells. This concept was first proposed by Schofield in 1978 for the hematopoietic cell system [59]. According to this hypothesis, stem cells cannot survive long enough to function as SSCs outside of the basal compartment niche. Therefore, a stem cell needs this niche to proliferate in controlled manner and to execute its role in the body. There might be several important players in SSC niche formation: Sertoli cells, the basement membrane, peritubular myoid cells, and undefined signals external to the seminiferous tubules. Sertoli cells might be the most important component, as they provide growth factors for SSCs and have been described to insinuate themselves between all of the neighboring germ cells, leaving very few regions with evident contact between germ cells. Recent studies have demonstrated that Sertoli-cell-derived growth factor, glial-cell-line-derived neurotrophic factor, plays a key role to promote SSC survival and self-renewal, thereby stimulating SSC proliferation *in vivo* and *in vitro* [60–64]. rAT-MSCs, injected in the testes, were localized both in and out of the seminiferous tubules, from which SSCs were removed by busulfan treatment. As soon as the migrated MSCs had contact with the niche in the seminiferous tubules and with the cytokines secreted by the Sertoli cells, they might transdifferentiated into SSCs and initiate the spermatogenesis. This specific microenvironment could provide a unique and excellent condition for meiosis and functional sperm formation to MSCs. Therefore, the rAT-MSCs demonstrated a better differentiation into functional sperm compared to the *in vitro* differentiation studies.

 The main handicap of the busulfan treatment in rat models was the possibility of severe destruction of niche, especially Sertoli cells in testes. This damage could be the explanation of why the spermatogenesis was not started in all seminiferous tubules, but the activity was limited only in a couple of tubules. The destruction of Sertoli cells by busulfan treatment caused the damaged of niche, such that it did not have the capacity to support the transdifferentiation of migrated rAT-MSCs anymore. It was considered that the niche might play a vital role in differentiation of rAT-MSCs into functional sperm. 

 The differentiation of insufficient number of cells and the high number of cells in the phase of incomplete meiosis are considered the two main drawbacks of germ-like cell derivation of stem cells [64]. In those studies, small number of cells after busulfan treatment was positive for specific germ cell markers, like VASA and SCPs. The differentiation took place not in all but in some tubules. In our study, the busulfan treatment was proven to be effective in elimination of cells in tubules, but the overexposure might also cause the damage of niche. The spermatogenesis activity was only observed to start in a few tubules, but it was sufficient to initiate successful pregnancy. 

 The male rats recovered their fertility after receiving the injection of AT-MSCs. The stem cells could affect either by maintaining the preexisting SSCs leading to reinitiate the spermatogenesis or by transdifferentiation into SSC-like cells to form spermatocytes. To explain this event, the AT-MSCs were transferred with GFP gene before injection in testes, and the integrity of this gene into genome was assured. Following mating and pregnancy, the first generation of offspring was obtained. The sperms recovered from the offspring were GFP^+^. MSCs isolated from the tissues of offspring were analyzed, and GFP was shown to be expressed in cells by immunostaining. Although this exogenous gene was integrated into genomic DNA, GFP expression was not observed in all cells of offspring. The GFP gene was transferred for reporting purpose and not for generating a transgenic animal. The vector was designed to express GFP gene in cells under the control of CMV promoter. Hypermethylation of the viral promoter sequences by de novo DNA methylation in host cells might cause silencing of the transgene in some cells and in next generations [65]. But the important point is that the only origin of GFP could be the rAT-MSCs injected into the testes. The results indicated that these cells transdifferentiated into functional sperm in seminiferous tubules, and these sperms later involved in delivery of GFP gene to offspring genome. As the sperm formation was not observed *in vitro* and also in severely damaged tubules, it might point to the significance of the well-preserved niche for the differentiation MSCs into sperm. There is also another possibility, which should be taken into account, that the MSCs had the characteristics to fuse with other cells spontaneously [66]. From the early studies with MSCs, it was well known that these cells could fuse with somatic cells, albeit rarely. It was found that the frequency of fusion between MSCs and differentiated cells increased in the presence of TNF-alpha and/or IFN-gamma [67]. The apoptosis was induced in testes by busulfan, and these cytokines were highly expressed, consequently. The increase in the fusion events was expected in testes. It should be noticed that there was a period of 12 weeks between the busulfan treatment and MSCs injection, and the inflammatory factors were reduced during this period. For spermatogenesis activity, MSCs should form hybrid cell with existing SSCs. However, the severe damage by busulfan eliminated almost all SSCs in tubules, and the only cells left were Sertoli cells. Although they are very important for niche, they could not have the capacity for spermatogenesis. Therefore, the fusion could not have played a significant role in the recovery of infertility.

On the other hand, the expression of pluripotency markers by rAT-MSCs might have an effect on sperm generation. Although the mesenchymal stem cells are generally classified as multipotent with respect to their differentiation capacity, the expression of these pluripotency markers could enhanced their differentiation potential into various tissues [68]. Obviously, it was not enough for sperm generation from MSCs in cell culture. Some additional factors might be required that could be provided by niche. The expression levels of some pluripotency genes (Oct4, Sox2, Rex1, and FoxD3) were analyzed for the same rAT-MSC line (unpublished data). The expression for Sox2 and FoxD3 could not be detected, but there was relatively strong expression of Rex1 and weak expression of Oct4. The expression of Rex1 is known to preserve the undifferentiated state of stem cells. This gene was also suggested to be important in spermatocytes, and it might play a role in meiosis [69]. Beside the effect of niche, the intrinsic factors, like expression of Rex1, might also have direct or indirect effects on sperm generation.

In conclusion, the fertile status of busulfan treated-male rats was recovered by rAT-MSCs transplantation in this study. The GFP^+^ cells were found both outside of the basal compartment and in the seminiferous tubules, supporting the idea that MSCs might have functioned in reestablishment of spermatogenesis by two ways: MSCs' differentiation into sperm, or maintenance of SSCs. These results showed the rAT-MSCs could be both rich and functional source for the infertility treatment. The most important issue of this study was the achievement of first successful results in spermatogenesis by endogen reprogramming using adult stem cells. If this protocol was also proven to be functional in human, the possibility to treat the males with azoospermia would arouse.

## Supplementary Material

Supplementary Figure 1: Immunostaining of undifferentiated rAT-MSCs for VASA and SCP1. The expression of meiotic (SCP1) and spermatogenic cell markers (VASA) was not observed in stem cell cultures in vitro. DAPI was used for nuclei staining (blue). Scale bars: 50*µ*m.
Supplementary Figure 2: Immunostaining of rAT-MSCs for GFP. GFP labeled rAT-MSCs were stained with antibody to GFP (Santa Cruz, sc-5385) (A1-A3). The staining pattern of GFP+ MSCs was cytoplasmic, but most of the luminescence was observed around the nuclei. The GFP staining of rAT-MSCs were compared with the negative control, untransformed rAT-MSCs (B1-B3).
Supplementary Figure 3: GFP*+* sperms from offspring. Sperms from offspring collected on glass slides by the cytocentrifuge were stained with GFP antibody (A1-A3). The nuclei were labeled with DAPI. The control slides were stained with secondary antibody and DAPI, to show the autofluorescence (B1-B3). Scale bars: 20*µ*m.

## Figures and Tables

**Figure 7:**
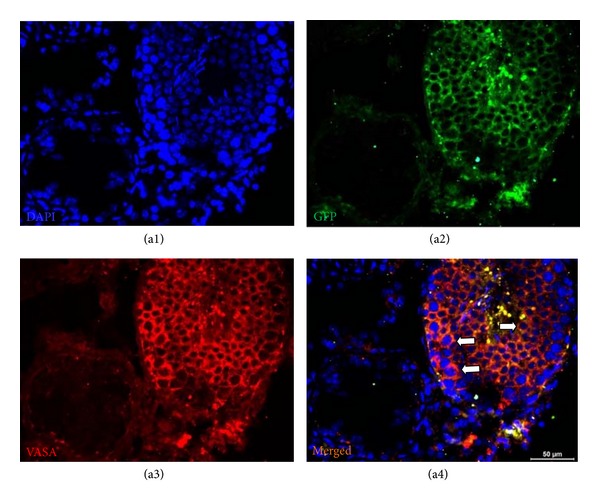
GFP^+^ cells in seminiferous tubule. MSC-received testis was fixed and stained for GFP (green) and spermatogenic cell marker, VASA (red). The cells in seminiferous tubule with dual staining were shown by arrows (a4). The adjacent tubules showed no staining for VASA, which indicated the absence of spermatogenic activity.

**Table 1:** Immunocytochemical properties of rAT-MSCs.

Antibody/marker	Dilution	Source	Detection
CD 26	1 : 50	Santa Cruz Bio.	+
CD 34 (C-18)	1 : 150	Santa Cruz Bio.	*∅*
CD 45 (H-230)	1 : 150	Santa Cruz Bio.	*∅*
CD 71 (K-20)	1 : 150	Santa Cruz Bio.	*∅*
CD105/Endoglin (M-20)	1 : 100	Santa Cruz Bio.	+
c-Fos (4)	1 : 50	Santa Cruz Bio.	+
Collagen II (2B1.5)	Predilute	Thermo Scientific	+
Collagen Ia1 (D-13)	1 : 50	Santa Cruz Bio.	+
*β*-tubulin	1 : 50	Santa Cruz Bio.	+
Nestin (Rat-401)	1 : 50	Santa Cruz Bio.	+
Vimentin (C-20)	1 : 100	Santa Cruz Bio.	+
Fibronectin (EP5)	1 : 100	Santa Cruz Bio.	+
ASMA	1 : 800	Thermo Scientific	+
Myogenin (F5D)	Predilute	Thermo Scientific	+
MAP 2a, b (AP20)	Predilute	Thermo Scientific	+
GFAP	Predilute	Thermo Scientific	+
Osteocalcin (FL-100)	1 : 50	Santa Cruz Bio.	+
Osteonectin (SPARC)	1 : 50	Millipore	+
Osteopontin (AKm2A1)	1 : 50	Santa Cruz Bio.	+
Ki67	1 : 300	Thermo Scientific	+
PCNA	1 : 200	Thermo Scientific	+
Tenascin-C	1 : 50	Santa Cruz Bio.	+
Cytokeratin 18	1 : 50	Santa Cruz Bio.	*∅*

+: positive; *∅*: lack of marker expression.
