# Supplementary material for: Recovery of Fertility in Azoospermia Rats after Injection of Adipose-Tissue-Derived Mesenchymal Stem Cells: The Sperm Generation
Source: Biomed Res Int. 2013 Feb 18;2013:529589. doi: 10.1155/2013/529589 (PMC3590610; doi:10.1155/2013/529589)
Supplement: Supplementary file 1 — Supplementary Figure 1: Immunostaining of undifferentiated rAT-MSCs for VASA and SCP1. The expression of meiotic (SCP1) and spermatogenic cell markers (VASA) was not observed in stem cell cultures in vitro. DAPI was used for nuclei staining (blue). Scale bars: 50µm. Supplementary Figure 2: Immunostaining of rAT-MSCs for GFP. GFP labeled rAT-MSCs were stained with antibody to GFP (Santa Cruz, sc-5385) (A1-A3). The staining pattern of GFP+ MSCs was cytoplasmic, but most of the luminescence was observed around the nuclei. The GFP staining of rAT-MSCs were compared with the negative control, untransformed rAT-MSCs (B1-B3). Supplementary Figure 3: GFP+ sperms from offspring. Sperms from offspring collected on glass slides by the cytocentrifuge were stained with GFP antibody (A1-A3). The nuclei were labeled with DAPI. The control slides were stained with secondary antibody and DAPI, to show the autofluorescence (B1-B3). Scale bars: 20µm. [file 529589.f1.doc]

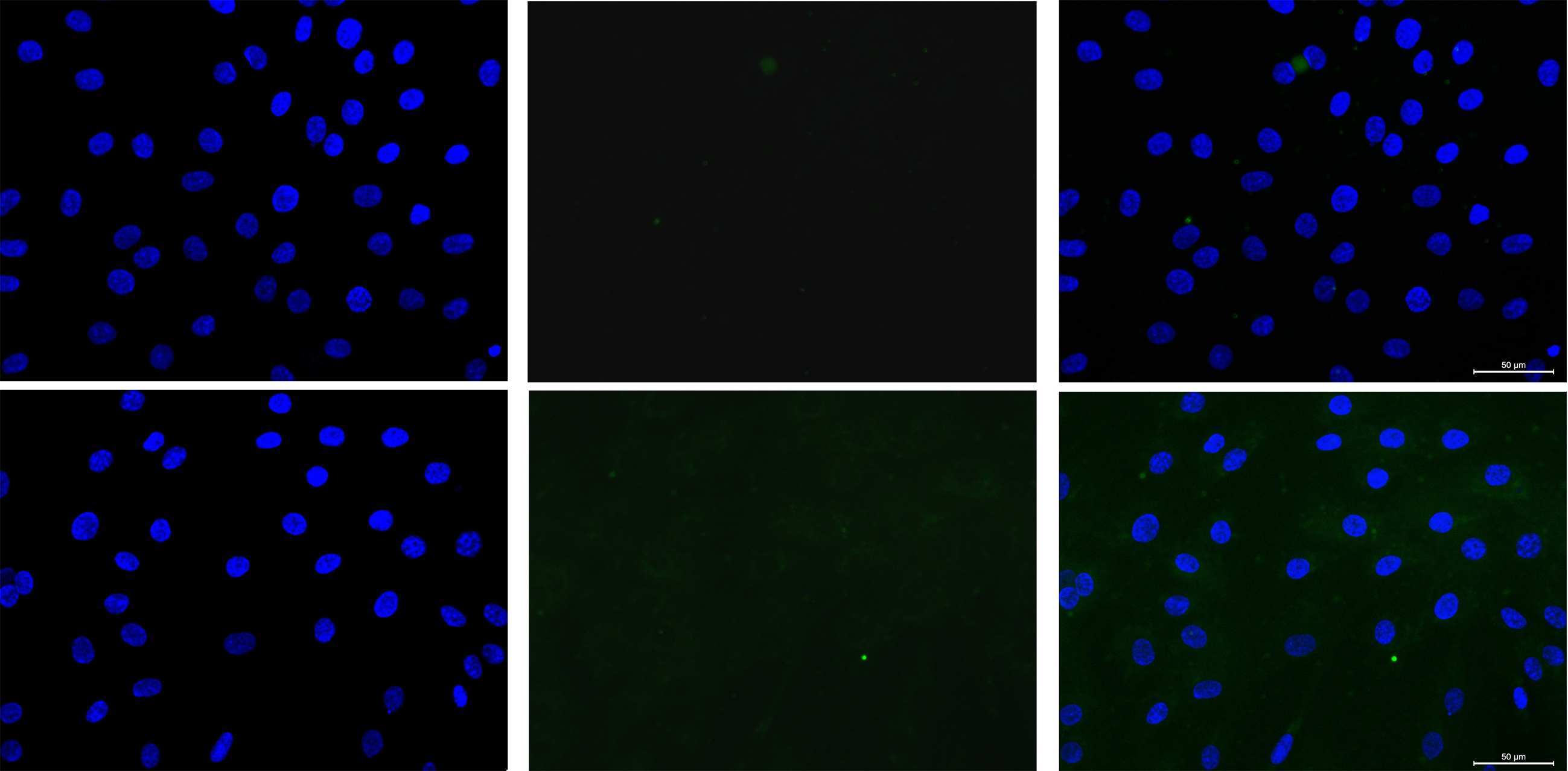


**DAPI**

**DAPI**

**SCP-1**

**VASA**

**merged**

**merged**

**SUPPLEMENTARY FIGURE 1.** Immunostaining of undifferentiated rAT-MSCs for VASA and SCP1. The expression of meiotic (SCP1) and spermatogenic cell markers (VASA) was not observed in stem cell cultures *in vitro*. DAPI was used for nuclei staining (blue). Scale bars: 50μm.
