# Supplementary material for: Recovery of Fertility in Azoospermia Rats after Injection of Adipose-Tissue-Derived Mesenchymal Stem Cells: The Sperm Generation
Source: Biomed Res Int. 2013 Feb 18;2013:529589. doi: 10.1155/2013/529589 (PMC3590610; doi:10.1155/2013/529589)
Supplement: Supplementary file 2 [file 529589.f2.doc]

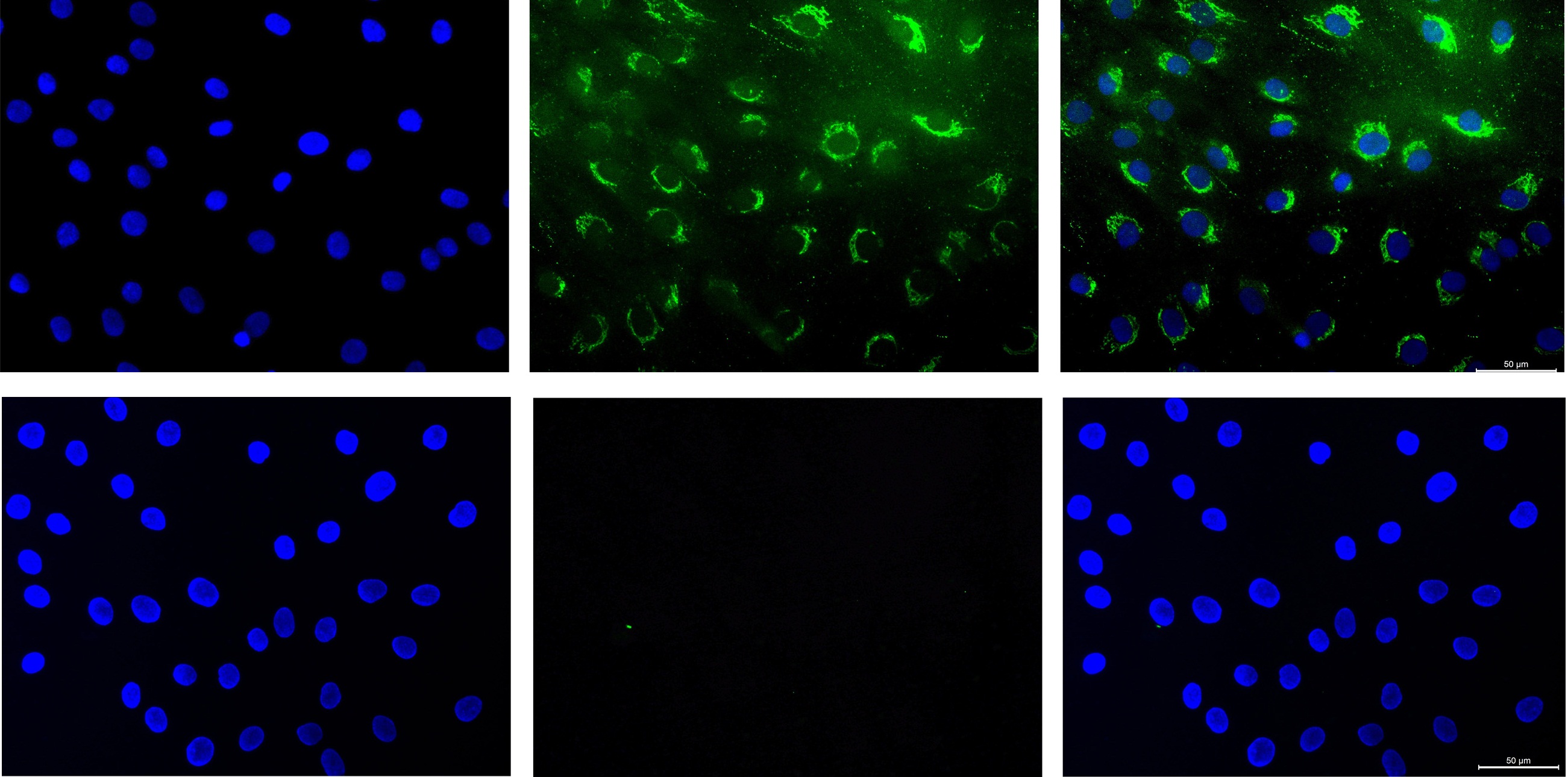


**A1**

**A2**

**B1**

**B2**

**A3**

**B3**

**DAPI**

**DAPI**

**GFP**

**GFP**

**merged**

**merged**

**SUPPLEMENTARY FIGURE 2.** Immunostaining of rAT-MSCs for GFP. GFP labeled rAT-MSCs were stained with antibody to GFP (Santa Cruz, sc-5385) (**A1-A3**). The staining pattern of GFP+ MSCs was cytoplasmic, but most of the luminescence was observed around the nuclei. The GFP staining of rAT-MSCs were compared with the negative control, untransformed rAT-MSCs (**B1-B3**).
