# Supplementary material for: Recovery of Fertility in Azoospermia Rats after Injection of Adipose-Tissue-Derived Mesenchymal Stem Cells: The Sperm Generation
Source: Biomed Res Int. 2013 Feb 18;2013:529589. doi: 10.1155/2013/529589 (PMC3590610; doi:10.1155/2013/529589)
Supplement: Supplementary file 3 [file 529589.f3.doc]

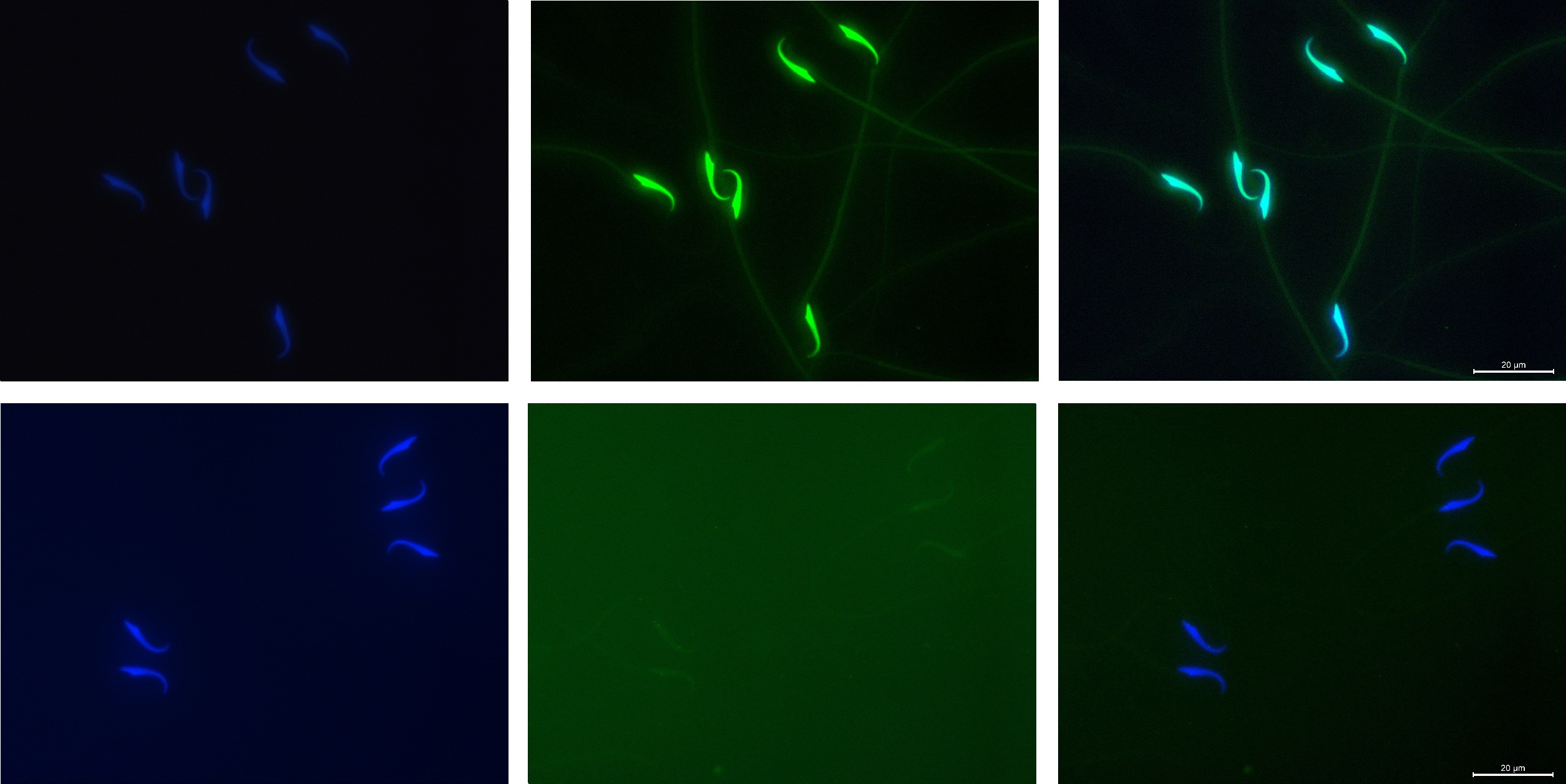


**A1**

**A2**

**B1**

**B2**

**A3**

**B3**

**DAPI**

**DAPI**

**GFP**

**GFP**

**merged**

**merged**

**SUPPLEMENTARY FIGURE 3.** GFP+ sperms from offspring. Sperms from offspring collected on glass slides by the cytocentrifuge were stained with GFP antibody (**A1-A3**). The nuclei were labeled with DAPI. The control slides were stained with secondary antibody and DAPI, to show the autofluorescence (**B1-B3**). Scale bars: 20μm.
